# Transcriptional factor snail controls tumor neovascularization, growth and metastasis in mouse model of human ovarian carcinoma

**DOI:** 10.1186/s40169-014-0028-z

**Published:** 2014-09-23

**Authors:** Samar Abdulkhalek, Olivia D Geen, Lacey Brodhagen, Fiona Haxho, Farah Alghamdi, Stephanie Allison, Duncan J Simmons, Leah K O'Shea, Ronald J Neufeld, Myron R Szewczuk

**Affiliations:** Departments of Biomedical and Molecular Sciences, Queen's University, Kingston, K7L 3N6 ON Canada; Chemical Engineering, Queen's University, Kingston, K7L 3N6 ON Canada; Present address: Department of Molecular Genetics, Lerner Research Institute, Cleveland Clinic, Cleveland, Ohio USA; Present address: The King Fahd Armed Forces Hospital, Serology, Jeddah Saudi Arabia; Present address: Mississauga Academy of Medicine, University of Toronto Mississauga, North Terrence Donnelly Health Sciences Complex, Mississauga, L5L 1C6 ON Canada

**Keywords:** Human ovarian cancer, Tumor neovascularization, Silencing transcriptional repressors Snail and Slug, Oseltamivir phosphate

## Abstract

**Background:**

Snail, a transcriptional factor and repressor of E-cadherin is well known for its role in cellular invasion. It can regulate epithelial to mesenchymal transition (EMT) during embryonic development and in epithelial cells. Snail also mediates tumor progression and metastases. Silencing of Snail and its associate member Slug in human A2780 ovarian epithelial carcinoma cell line was investigated to identify its role in tumor neovascularization.

**Methods:**

Live cell sialidase, WST-1 cell viability and immunohistochemistry assays were used to evaluate sialidase activity, cell survival and the expression levels of tumor E-cadherin, N-cadherin, VE-cadherin, and host endothelial CD31+(PECAM-1) cells in archived paraffin-embedded ovarian A2780, A2780 Snail shRNA GIPZ lentiviral knockdown (KD) and A2780 Slug shRNA GIPZ lentiviral KD tumors grown in RAGxCγ double mutant mice.

**Results:**

Oseltamivir phosphate (OP), anti-Neu1 antibodies and MMP-9 specific inhibitor blocked Neu1 activity associated with epidermal growth factor (EGF) stimulated A2780 ovarian epithelial carcinoma cells. Silencing Snail in A2780 cells abrogated the Neu1 activity following EGF stimulation of the cells compared to A2780 and A2780 Slug KD cells. OP treatment of A2780 and cisplatin-resistant A2780cis cells reproducibly and dose-dependently abated the cell viability with a LD_50_ of 7 and 4 μm, respectively, after 48 h of incubation. Heterotopic xenografts of A2780 and A2780 Slug KD tumors developed robust and bloody tumor vascularization in RAG2xCγ double mutant mice. OP treatment at 50 mg/kg daily intraperitoneally did not significantly impede A2780 tumor growth rate but did cause a significant reduction of lung metastases compared with the untreated and OP 30mg/kg cohorts. Silencing Snail in A2780 tumor cells completely abrogated tumor vascularization, tumor growth and spread to the lungs in RAGxCγ double mutant mice. A2780 and A2780 Slug KD tumors expressed high levels of human N- and VE-cadherins, and host CD31+ endothelial cells, while A2780 Snail KD tumors expressed E-cadherin and reduced host CD31+ cells. OP 50mg/kg cohort tumors had reduced numbers of host CD31+ cells compared to a higher expression levels of CD31+ cells in tumors from the untreated control and OP 30mg/kg cohorts.

**Conclusion:**

Snail transcriptional factor is an important intermediate player in human ovarian tumor neovascularization.

**Electronic supplementary material:**

The online version of this article (doi:10.1186/s40169-014-0028-z) contains supplementary material, which is available to authorized users.

## Background

Epithelial ovarian cancer is a major cause of gynecologic cancer-associated deaths [1]. Patients with ovarian cancer have a low 5-year survival with frequent recurrences and rapid metastases to the peritoneal cavity with concomitant malignant pleural effusions. The increased invasiveness and motility of ovarian tumor cells are facilitated by several molecular players that involve epithelial-mesenchymal transition (EMT) [2]-[5]. One of the major transitions of EMT is the loss of E-cadherin expression which is a cell-cell adhesion molecule that participates in homotypic, calcium-dependent interactions to form epithelial adherens junctions [6]. Loss of E cadherin expression is well correlated with tumor grade and cancer stage. Several molecular mechanisms underlying loss of E-cadherin expression have been identified. For example, hyper-methylation of the E-cadherin promoter and alterations in transcription, but not mutations have emerged as one of the major mechanisms responsible for E-cadherin down-regulation in most carcinomas [7]. In addition, several transcriptional repressors of E-cadherin have been identified, including the zinc-finger factors Snail and Slug, the two-handed zinc factors ZEB-1/dEF1 and ZEB-2/SIP1 and the basic helix-loop-helix transcription factors Twist and E12/E47 (the E2A gene product). Epithelial cells that ectopically express E47 adopt a fibroblastic morphology that develops tumorigenic and migratory/invasive properties with a concomitant loss of E-cadherin. The integration of these transcriptional suppressor families in epithelial carcinoma plasticity is eloquently reviewed by Grunert et al. [2] and Cano and colleagues [8].

Unlike most carcinomas that dedifferentiate during neoplastic progression with loss of epithelial E-cadherin, ovarian carcinomas undergo a transition to a more epithelial phenotype, early in tumor progression, with increased E-cadherin expression. Subsequent reacquisition of mesenchymal features is observed in late stage tumors, and a loss of E-cadherin expression or function is an observed feature in ovarian cancer progression [9]-[11]. Indeed, it has been reported that epidermal growth factor (EGF) receptor (EGFR) is frequently elevated in epithelial ovarian cancer, and that E-cadherin expression is often reduced in the advanced stages of the disease [12]. Hudson and colleagues have shown that EGFR activation promotes a phosphatidylinositol 3-kinase (PI3K)-dependent induction of a cell surface pro-MMP-9 binding component that facilitates gelatinase-mediated cellular invasion [13]. This EGFR activation was shown to promote disruption of adherens junctions through induction of MMP-9. Stable overexpression of MMP-9 led to a loss of E-cadherin and junctional integrity, and promoted a migratory and invasive phenotype in ovarian cancer. Others have reported additional confirmation that the expression of zinc-finger factor Snail is associated with an increase in the promoter activity and expression of MMP-9, and that the induced transcription of MMP-9 by Snail is driven by a mechanism dependent on the MAPK and PI3K signaling pathways [14]. Collectively, these findings indicate that MMP-9 transcription is activated in response to Snail expression and that it might explain, in part, the invasive properties of the Snail-expressing cells. The MMP-Snail signaling axis and its functional role in tumor progression with an insight into new anti-cancer therapeutic strategies is eloquently reviewed Przybylo and Radisky [15].

Cancer cells utilize signaling initiated by EGFRs to establish an anti-apoptotic state within the cell as well as to up-regulate mitogenic, angiogenic and pro-invasive cellular mechanisms [16]. The activation of the EGF receptors regulates many processes associated with metastasis, including modulation of cell-cell and cell-substrate interactions, production of matrix-degrading proteinases, and cellular migration. EGFR signaling has also been linked to EMT [17]. For an example, therapeutic strategies targeting the EGF receptors and their inhibition caused a reversal of EMT in human pancreatic cancer [17]. Other studies have suggested the potential role of growth factor receptor signaling in establishing chemo-resistance in cancer cells and tumors [18]-[22]. Signals derived from the cellular microenvironment can also regulate EMT, such as cell-cell contacts mediated by families of transmembrane receptors and ligands expressed on adjacent cells [23]. At the genetic level, several transcriptional suppressor families are known that regulate EMT, including the zinc-finger proteins Snail1 (Snail) and Snail2 (Slug), the two-handed zinc-finger δEF1 family factors (δEF1/Zeb1 and SIP1/Zeb2), and the basic helix-loop-helix factors, Twist and E12/E47 [23]. What is less clear is the direct relationship between EMT and transcriptional suppressor factors in tumor neovascularization.

An insight into the mechanism of EGF-induced receptor activation came from our recent report on Neu1 sialidase and MMP-9 cross-talk in regulating EGF receptors [24]. The report discloses a receptor signaling paradigm involving an EGF-induced G-protein coupled receptor (GPCR)-signaling process and MMP-9 activation to induce Neu1. This tripartite complex of neuromedin B GPCR, MMP-9 and Neu1 forms an alliance with EGFR tethered at the ectodomain of the receptor on the cell surface. Active Neu1 in complex with EGFR hydrolyzes α-2,3-sialyl residues of the receptors, enabling the removal of steric hindrance to receptor association and allowing subsequent dimerization, activation, and cellular signaling. A striking similarity of this novel receptor signaling platform was reported by us for nerve growth factor (NGF) TrkA receptors [25], insulin receptors [26], cell surface TOLL-like receptor-4 (TLR-4) [27]-[31] and intracellular TLR-7 and -9 [32]; these receptors are known to play major roles in cancer. Oseltamivir phosphate (OP, Tamiflu®) was found to specifically target and inhibit Neu1 activity associated with these ligand-induced receptor activations [25],[31],[33]. The findings in the report for EGF receptors also propose an alternate therapeutic approach using OP as an anti-cancer agent targeting Neu1 sialidase as the key central enzyme within this novel EGFR signaling platform. Preclinical molecular-targeting studies provide the proof-of-evidence for an effective OP therapy in the treatment of human pancreatic cancer growth and metastatic spread in heterotopic xenograft of tumors growing in RAGxCγ double mutant mice [24]. In addition, OP overcame the chemo-resistance of pancreatic cancer PANC1 cells to cisplatin and gemcitabine alone or in combination in a dose-dependent manner, and disabled the cancer cell survival mechanism(s) against the chemotherapeutic drugs [22]. More interestingly, the data in the report also provided additional confirmation for OP therapy in reversing EMT characteristic of E- to N-cadherin changes associated with resistance to standard, clinical chemotherapeutics.

This present report describes another molecular level of a novel organizational signaling platform connecting the Snail-MMP-9 signaling axis in amplifying the Neu1 sialidase and MMP-9 cross-talk in regulating EGF receptors, tumor neovascularization, growth and invasiveness.

## Methods

### Cell lines

A2780 (human ovarian epithelial carcinoma, Sigma-Aldrich cat # 93112519**)** was established from tumor tissue from an untreated patient. A2780 is the parent line to the cisplatin resistant cell line A2780cis (Sigma-Aldrich cat # 93112517). The cells were grown in a 5% CO_2_ incubator at 37°C in culture containing Dulbecco's Modified Eagle's Medium (Gibco, Rockville, MD, USA) supplemented with 10% fetal calf serum (HyClone, Logan, UT, USA) and 5 μg/mL Plasmocin™ (InvivoGen, San Diego, CA, USA). When the cells reached ∼80% confluence, they were passaged at least five times for use in the experiments.

### Silencing Snail and Slug mRNA in A2780 cells using GIPZ lentiviral shRNA transfection particles

Human Snail (gene symbol SNAIL1, V3LHS-32872 at 1.7 × 10^8^ transducing unit (TU)/mL) and Slug (gene symbol SNAI2, V3LHS-390965 at 3.98 × 10^8^ TU/mL) pGIPZ lentiviral shRNA ready-to-use particles (based on miR-30 for gene knockdown) were obtained from Thermo Scientific (Open Biosystems). The GIPZ Lentiviral shRNA platform minimizes the potential generation of recombinant viruses to the highest level by combining a disabled viral genome with the proprietary Trans-Lentiviral packaging process. The protocol uses a third generation self-inactivating packaging system meeting BioSafety Level 2 requirements with the University Biohazard committee approval. The GIPZ Lentifect particles include a CMV promoter for efficient expression of non-tagged eGFP in target cells and use a puromycin resistance marker for selection of stably transduced cells. Ready-to-use lentiviral particles were used for the transduction of A2780 ovarian carcinoma cells. Briefly, cells were cultured in 6 well tissue culture plates in DMEM medium containing 10% FCS and 5 μg/mL plasmocin. After 24 h, medium was discarded and 2 mL of 5 μg/mL of polybrene media was added to the cells followed by GIPZ lentiviral shRNA particles at MOI = 6. The plate was mixed, centrifuged at 2500 rpm for 90 min and incubated at 37°C in 5% CO_2_ humidified incubator for 24 h. The cells were washed and re-cultured in media for an additional 2 days. On day 5, the media were replaced with selection media containing optimal 2 μg/mL of puromycin as pre-determined in a cell viability assay. Selection media was added every 40h to expand puromycin-resistant Snail shRNA and Slug shRNA transduced A2780 cell clones. The transfection efficiency was determined to be 99% knockdown for both Snail and Slug using RT-PCR analyses of total RNAs from parental A2780, Snail shRNA and Slug shRNA knockdown cells.

### Reagents

Epidermal growth factor (EGF; Sigma-Aldrich, St. Louis, MO), the natural ligand of the EGF receptor, was reconstituted in sterile 1× phosphate buffered saline (PBS) at a stock concentration of 1 mg/mL and stored at −20°C. EGF concentrations to stimulate cells were 30–100 ng/mL. Incubation times vary between experiments and thus are indicated.

*cis*-Diamineplatinum(II) dichloride (P4394, Sigma-Aldrich Canada Ltd) was dissolved in dimethyl sulfoxide (DMSO) to create a 27.7mM cisplatin stock. Gemcitabine hydrochloride (G6423, Sigma-Aldrich Canada Ltd) was dissolved in phosphate-buffered saline (PBS) to create a 133.5 mM gemcitabine stock. 5-Fluorouracil (5-FU) (F6627, Sigma-Aldrich Canada Ltd) was dissolved 1 mL DMSO plus 9 mL PBS to create a 2.31 mM 5-FU stock. Paclitaxel from *Taxus brevifolia*, ≥95% (HPLC) (T7402, Sigma-Aldrich Canada Ltd) was dissolved in DMSO to create a 1.17 mM paclitaxel stock. These stocks were serially diluted in 1 × Dulbecco's Modified Eagle's Medium (10% fetal calf serum and 5 μg/mL Plasmocin). The stock solutions were further diluted in the medium to make various dosages of the chemotherapeutic agents to be used in the in vitro experiments.

### Inhibitors

Oseltamivir phosphate (OP) free base (Hoffmann-La Roche Ltd, Mississauga, ON, Canada) was used at the concentrations indicated. OP 75 mg capsules were dissolved in sterile phosphate-buffered saline and centrifuged at 1,000 rpm for 10 minutes to remove the filler as previously reported by us [22]. The stock-extracted oseltamivir phosphate solution had a concentration of 15 mg/mL. Cell culture medium containing 1 × Dulbecco's Modified Eagle's Medium, 10% fetal calf serum, and 5 μg/mL Plasmocin with different concentrations of oseltamivir phosphate (200-800 μg/mL) were used for the in vitro and in vivo experiments. MMP-9 inhibitor (MMP-9 Inhibitor-I, CAS1177749-58-4, Calbiochem-EMD Chemicals Inc.) is a cell-permeable, potent, selective, and reversible MMP-9 inhibitor (IC_50_ = 5 nM). It also inhibits MMP-1 (IC_50_ = 1.05 m) and MMP-13 (IC_50_ = 113 nM) only at much higher concentrations.

### Primary antibodies

Neutralizing antibodies were used to inhibit sialidase function: rabbit anti-human Neu1 IgG antibody (Santa Cruz Biotechnology, Santa Cruz, CA, USA).

Rabbit monoclonal E-cadherin antibody serum (Cell Signaling Technology, Inc, Danvers, MA, USA), which recognizes the human E-cadherin epitope, was used for immunohistochemistry using a 1:400 dilution according to the manufacturer's instructions. Purified rabbit monoclonal antibody against human N-cadherin (Cell Signaling Technology, Inc.) and human VE-cadherin (Cell Signaling Technology, Inc.) epitopes, respectively, were used in immunohistochemistry at a 1:200 dilution according to the manufacturer's instructions.

DyLight™ 488 donkey anti-rabbit IgG secondary antibody (Santa Cruz Biotechnology, Inc, Santa Cruz, CA, USA) was used at a final concentration of 40 μg/mL for immunohistochemistry to detect primary antibodies against human E-cadherin, N-cadherin and VE-cadherin in paraffin-embedded xenogafts of human A2780 ovarian tumors.

DyLight™ 488 rat monoclonal anti-mouse CD31 (PECAM-1) antibody (Novus Biologicals Canada ULC, Oakville ON L6M 2V5, Canada) was used in immunohistochemistry at a 1:300 dilution according to the manufacturer's instructions to detect mouse CD31+ (PECAM-1) endothelial cells in the paraffin-embedded xenogafts of A2780 ovarian tumors.

### Sialidase assay in live cells

Cells were grown overnight on 12 mm circular glass slides in conditioned medium in a sterile 24-well tissue plate until they reached ~70% confluence as previously optimized in the live cell sialidase assay [29],[31]. After removing medium, 0.318 mM 4-MUNANA (2′-(4-methylumbelliferyl)-α-D-N-acetylneuraminic acid; Biosynth Intl.) substrate in Tris-buffered saline (TBS) pH 7.4 was added to each well alone (control), with predetermined dose of EGF (30 ng/mL), or in combination of EGF and inhibitor or neutralizing antibodies at the indicated concentrations. The substrate is hydrolyzed by sialidase to give free 4-methylumbelliferone which has a fluorescence emission at 450 nm (blue color) following an excitation at 365 nm. Fluorescent images were taken after 1-2 min using Zeiss M2 Imager epifluorescent microscopy (Carl Zeiss AG, Oberkochen, Germany) (40× objective). The mean fluorescence surrounding the cells was quantified using the Image J program.

### Heterotopic xenograft mouse model of human ovarian cancer

An immunodeficient mouse model with a double mutation in the combining recombinase activating gene (RAG) and common cytokine receptor γ chain (Cγ) was used as a heterotopic xenograft mouse model of human ovarian cancer as previously reported by us for human pancreatic cancer [34]. The RAG1xCγ double mutant mice on a Nod genetic background are completely alymphoid (T-cell, B-cell, and NK-cell deficient), show no spontaneous tumor formation, and exhibit normal hematopoietic parameters [35]. Mice were generated by inter-crossing and were maintained in SPF isolators in the Animal Care Facility, Queen's University, Kingston, Ontario K7L 3N6, Canada. A colony was established in the animal facility. All mice were kept under sterile conditions in micro-isolators or air-filtered cages, and were provided with autoclaved food and water. All mice used in the studies were approved by the Animal Care Committee, Queen's University. They were used between 6 and 8 weeks of age.

### Cancer cell implantation in RAGxCγ double mutant xenograft mice

Parental A2780 and Snail- and Slug shRNA knockdown A2780 cells under puromycin-resistance selection clones were grown in 75 cm^2^ cell culture flask at 80% confluence. The cells were resuspended into solution using TrypLE Express (Gibco) and washed with sterile saline. The cell suspension was centrifuged for 3 min at 900 rpm, and the cell pellet suspended in sterile saline at a concentration of 5–10 × 10^6^ cells/mL for 0.5 × 10^6^ cell implantation cutaneously into the right back flank of the mouse. Tumor measurements were taken twice a week. Tumor volumes were determined by (width square/2) × length. At the endpoint of the experiment at day 32-33 or earlier due to skin lesions, mice were euthanized by cervical dislocation and live necropsy tumor, liver and lung were weighed, and paraffin embedded for hematoxylin and eosin (H&E) staining and immunostaining for CD31(PECAM-1) cells in tumor tissues followed with microscopic analysis using a Zeiss M2 Fluorescence microscope.

### Immunohistochemistry

Immunohistochemistry of necropsy tumors was used to determine the presence of the characteristic human epithelial marker, E-cadherin, human mesenchymal marker, N-cahderin, and the endothelial cell marker, VE-cadherin in human A2780 tumors removed at necropsy from tumor-bearing RAG2xCγ double mutant mice which had received various treatments (oseltamivirS phosphate 30 and 50 mg/kg, Slug shRNA KD, Snail shRNA KD or untreated). Processed tumors were embedded in paraffin blocks following necropsy in each experiment. Tumor sections (5 μm) were deparaffinized, heated for 8 minutes in citrate buffer for antigen retrieval, placed in 0.03% H_2_O_2_ for 30 minutes to block endogenous peroxidases, rinsed three times in phosphate-buffered saline, and blocked in 1% bovine serum albumin (Fisher Scientific Company) overnight at 4°C. Deparaffinized and processed tumor sections (5 μm) were treated with 4% bovine serum albumin in Tween-Tris buffered saline and immunostained with primary and secondary antibody over one-hour periods. The primary antibody contained 2μg/mL rabbit anti-E-cadherin, anti-N-cadherin or anti-VE-cadherin followed with secondary donkey anti-rabbit IgG Alexa Fluor 488. The stained cells covered with Entellan® rapid mounting media were observed using a Zeiss M2 fluorescence microscope at 200× magnification. In addition, paraffin-embedded tumors were analyzed for mouse endothelial marker, CD31/PECAM-1. The primary antibody at 1:300 dilution of a stock 1 mg/mL of rat monoclonal antibody conjugated with DyLight-488 fluorochrome against mouse CD31/PECAM-1 (Novus Biologicals Canada ULC, Oakville, ON L6M 2V5 Canada).

### WST-assay

The WST-1 assay, a measure of cell viability based on the reduction of a tetrazolium compound to a soluble derivative was used [36]. The absorbance recorded at 420 nm is directly proportional to the number of living cells in culture. At 80%-90% confluence, cells were added to 96-well micro-well plates at a density of 5,000 cells/well and incubated overnight. They were then exposed to increasing concentrations of OP or left untreated as controls for 24, 48, and 72 hours. Absorbance readings were taken at 0, 24, 48, and 72 hours by adding WST-1 (Roche Diagnostics Division de Hoffman La Roche Limitée, Laval-des-Rapides, QC, Canada) as a cell proliferation reagent to each well (10% WST-1 in Dulbecco's Modified Eagle's Medium) followed by incubation at 37°C for 2 hours before reading at the indicated time points. Cell viability was presented as a percentage of control, and illustrated as a bar graph using GraphPad Prism software (GraphPad Software, La Jolla, CA, USA). The following formula was used to determine cell viability as a percent of control for each time point and OP/chemotherapeutic drug concentration:

[(Absorbance of cells in given concentration of drug) – (Media absorbance)]/[(Absorbance of cells alone) – (Media absorbance)] × 100.

### Statistical analysis

Statistical analysis was carried out using GraphPad Prism. Results were compared by a one-way ANOVA at 95% confidence using Bonferroni's multiple comparison test or unpaired t-test.

## Results

### Neu1 sialidase activity is associated with epidermal growth factor (EGF) stimulation of A2780 ovarian carcinoma cells

**Figure 1:**
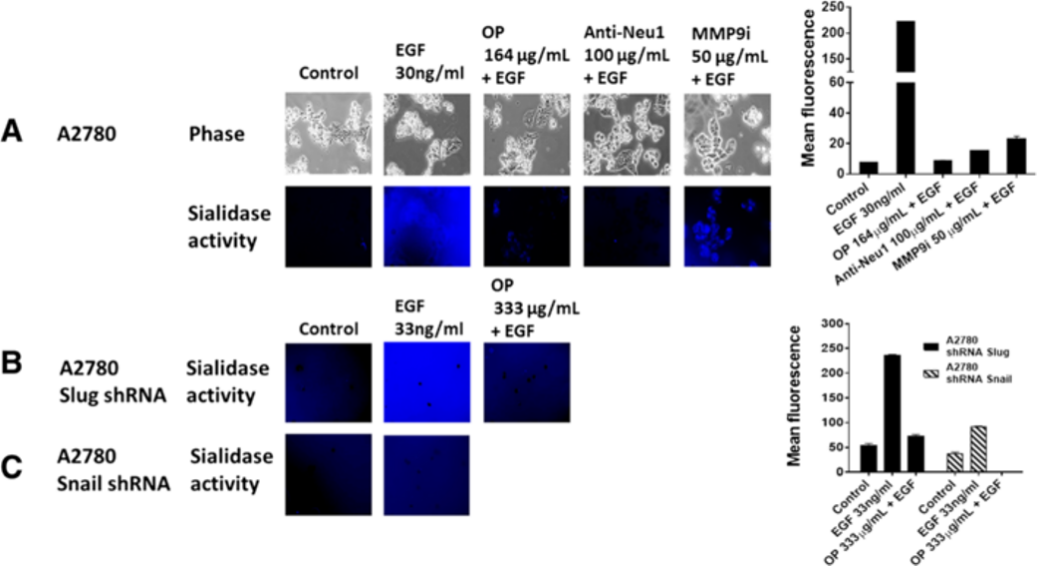
**EGF induces sialidase activity in live (A) A2780 ovarian cancer cells, (B) A2780 shRNA Snail and (C) A2780 shRNA Slug.** Cells were allowed to adhere on 12 mm circular glass slides in media containing 10% fetal calf sera for 24 h. After removing media, 0.318 mM 4-MUNANA substrate (2′-(4-methlyumbelliferyl)-α-N-acetylneuraminic acid) in Tris buffered saline pH 7.4 was added to live cells alone (control) or with EGF alone and in combination with OP, anti-Neu1 neutralizing antibodies, and specific inhibitor of MMP-9i at the indicated dosage. Fluorescent images were taken at 1-2 min after adding substrate using Zeiss Imager M2 epi-fluorescence microscopy (20× objective). The mean fluorescence of 50 multi-point replicates was quantified using the Image J software. The data are a representation of one out of three independent experiments showing similar results.

Since the co-expression of EGFR and MMP-9 has prognostic value in cancer [37],[38], and Neu1 and MMP-9 cross-talk regulates EGFRs in pancreatic cancer cells [34], we investigated the molecular targeting potential of the Neu1–MMP9 crosstalk platform in human ovarian cancer cell lines in vitro. Here, we performed a sialidase assay on the live cells as previously described [29],[31],[39]. As shown in Figure 1A, EGF stimulation of live A2780 cells induced sialidase activity. This sialidase activity is revealed in the periphery surrounding the cells using a fluorogenic sialidase specific substrate, 4-MUNANA [2′-(4-methylyumbelliferyl)-α-D-N-acetylneuraminic acid], which fluoresces at 450 nm caused by the emission of 4-methylumbelliferone. The mean fluorescence of 50 multi-point replicates was quantified using the Image J software and is depicted in the bar graph. We also used specific neutralizing antibodies against the human Neu1 as well as the neuraminidase inhibitor oseltamivir phosphate (OP) and a specific inhibitor of MMP9, all of which blocked the sialidase activity associated with EGF-treated live A2780 cells comparable to the levels of no EGF treated controls. The anti-Neu1 antibody used here is specific for the epitope corresponding to amino acids 116-415 mapping at the C-terminus of Neu1 of human origin. It also detects Neu1 of mouse, rat and human origin. The data depicted in Figure 1A are consistent with our previous report with EGF receptors in pancreatic cancers [34].

If Snail induces MMP-9 activation as previously reported [13],[14], we hypothesized that silencing Snail and its associated member Slug with shRNA lentiviral transfection of A2780 cells would indirectly block EGF-induced Neu1 sialidase activity at the ectodomain of the receptor. The data in Figure 1 support the hypothesis and provide additional evidence involving a role for Snail (Figure 1C) but not Slug (Figure 1B), indirectly regulating Neu1 sialidase activity associated with EGF-treated live A2780 cells.

### Viability of A2780, cisplatin-resistant A2780cis, A2780 shRNA Snail and A2780 shRNA Slug cells when treated with oseltamivir phosphate (OP) at different doses using the WST-1 assay

**Figure 2:**
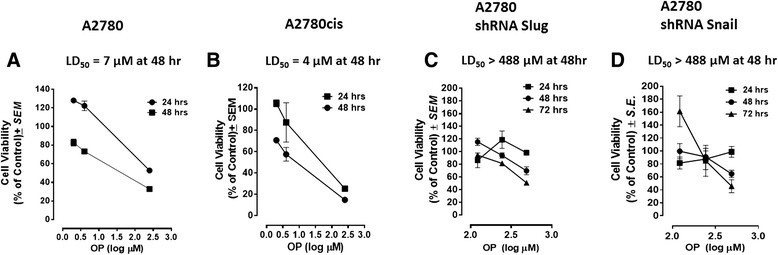
**Cell viability of (A) A2780, (B) A2780cis cells, (C) A2780 shRNA Slug and (D) A2780 shRNA Snail treated with OP at different doses using the WST-1 assay.** Cells were incubated in 96 well plates (5000 cells/well) and allowed to adhere for 24 h in 1× DMEM media containing 10% FCS. The media were replaced with fresh DMEM media containing 5% FCS without or with various concentrations of OP for indicated time periods. Cell viability was expressed as a percent of control ± *S.E.* of triplicate values. The data are a representation of one out of three independent experiments showing similar results. LD_50_ value is given as μm of drug concentration as determined by WST-1 assay after 48h for each of the cell lines.

To test the in vitro effects of OP on the cell viability of A2780 and cisplatin-resistant A2780cis cells, the cells were incubated in 96-well plates (5,000 cells/well) and allowed to adhere for 24 hours in 1 × Dulbecco's Modified Eagle's Medium containing 10% fetal calf serum. The medium was replaced with fresh Dulbecco's Modified Eagle's Medium containing 5% fetal calf serum without and with various concentrations of pure OP for 24 and 48 hours as predetermined. Cell viability as a percentage of control ± *S.E.* of triplicate values was determined using the WST-1 cell proliferation assay, which is a measure of cell viability based on the reduction of a tetrazolium compound to the soluble derivative [36]. The data shown in Figure 2A and 2B indicate that treatment of these ovarian cancer cell lines with OP reproducibly and dose-dependently decreased the cell viability (as a percentage of untreated control) with an LD_50_ of 7μm for A2780 (Figure 2A) and 4μm for A2780cis (Figure 2B) after 48 h of incubation. We also tested the in vitro effects of OP therapy on cell viability using the A2780 shRNA Snail and shRNA Slug cell clones. The data shown in Figure 2C and 2D indicate that OP treatment reproducibly and dose-dependently decreased the cell viability (as a percentage of untreated control) with an LD_50_ of >488μm for both A2780 shRNA Slug cells (Figure 2C) and A2780 shRNA Snail cells (Figure 2D) after 48h of incubation.

**Figure 3:**
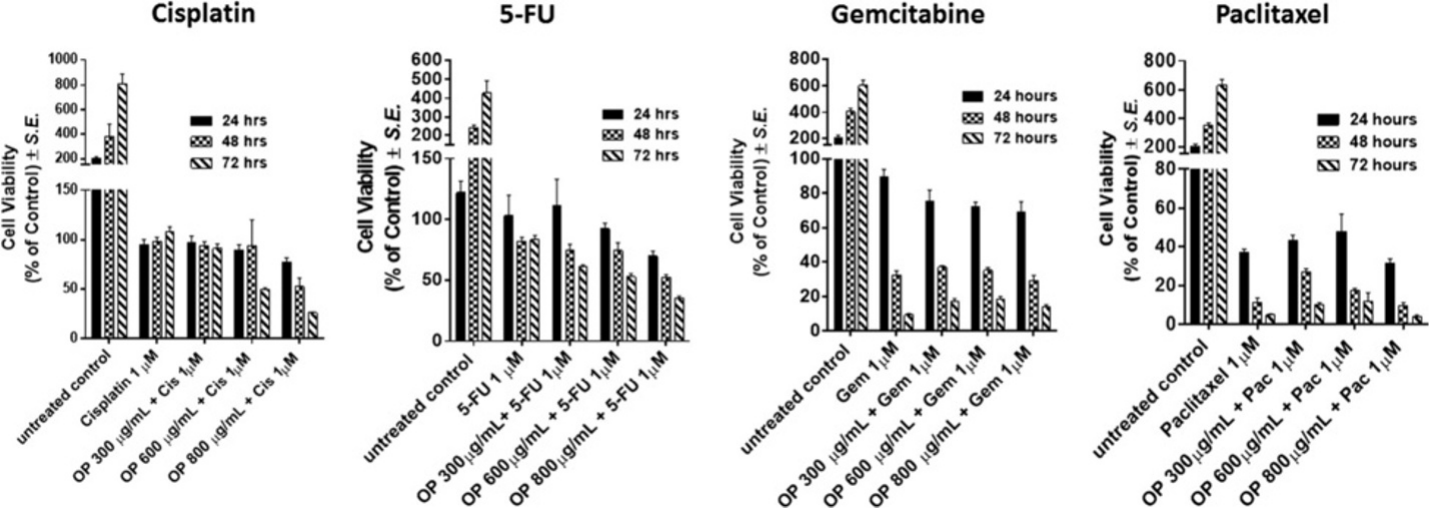
**Cell viability of A2780 cells treated with OP at indicated doses in combination with 1 μm of cisplatin, 5-FU, gemcitabine and paclitaxel using the WST-1 assay.** Cells were incubated in 96 well plates (5000 cells/well) and allowed to adhere for 24 h in 1× DMEM media containing 10% FCS. The media were replaced with fresh DMEM media containing 5% FCS without or with various concentrations of OP for 24, 48 and 72 hr as predetermined optimally. Cell viability was expressed as a percent of control ± *S.E.* of three independent experiments.

It is noteworthy that the OP LD_50_ value of 4μm for the cisplatin-resistant A2780cis cells was ∼2-fold lower than that for the parental A2780 cell line. These data are consistent with the results of our previous report indicating that treatment of long term chemo-resistance of PANC1 pancreatic cancer cells against 80μm cisplatin (PANC1-CisR) with OP caused a significant dose-dependent ∼96% reduction of cell viability [22]. It was hypothesized that cisplatin-resistant A2780cis cells in the presence of OP become more sensitive to the chemotherapeutic agent, resulting in decreased viability of the A2780cis cells. Using the WST-1 assay, the cell viability of A2780 cells treated with different dosages of OP in combination with 1μm of cisplatin, 5-FU, gemcitabine and paclitaxel was compared with that of the monotherapy of the chemo-drugs alone. The data in Figure 3 show that for the combination with cisplatin and 5-FU, only OP dosage ≥ 600μg/mL reduced cell viability at 72h compared to monotherapy, while OP does not apparently affect the activity of either gemcitabine or paclitaxel, when compared to the cell viability after single chemo-drug treatment.

### Targeting Neu1 with OP attenuates metastatic spread to lungs in heterotopic xenografts of A2780 cancer cells in RAGxCγ double mutant mice

**Figure 4:**
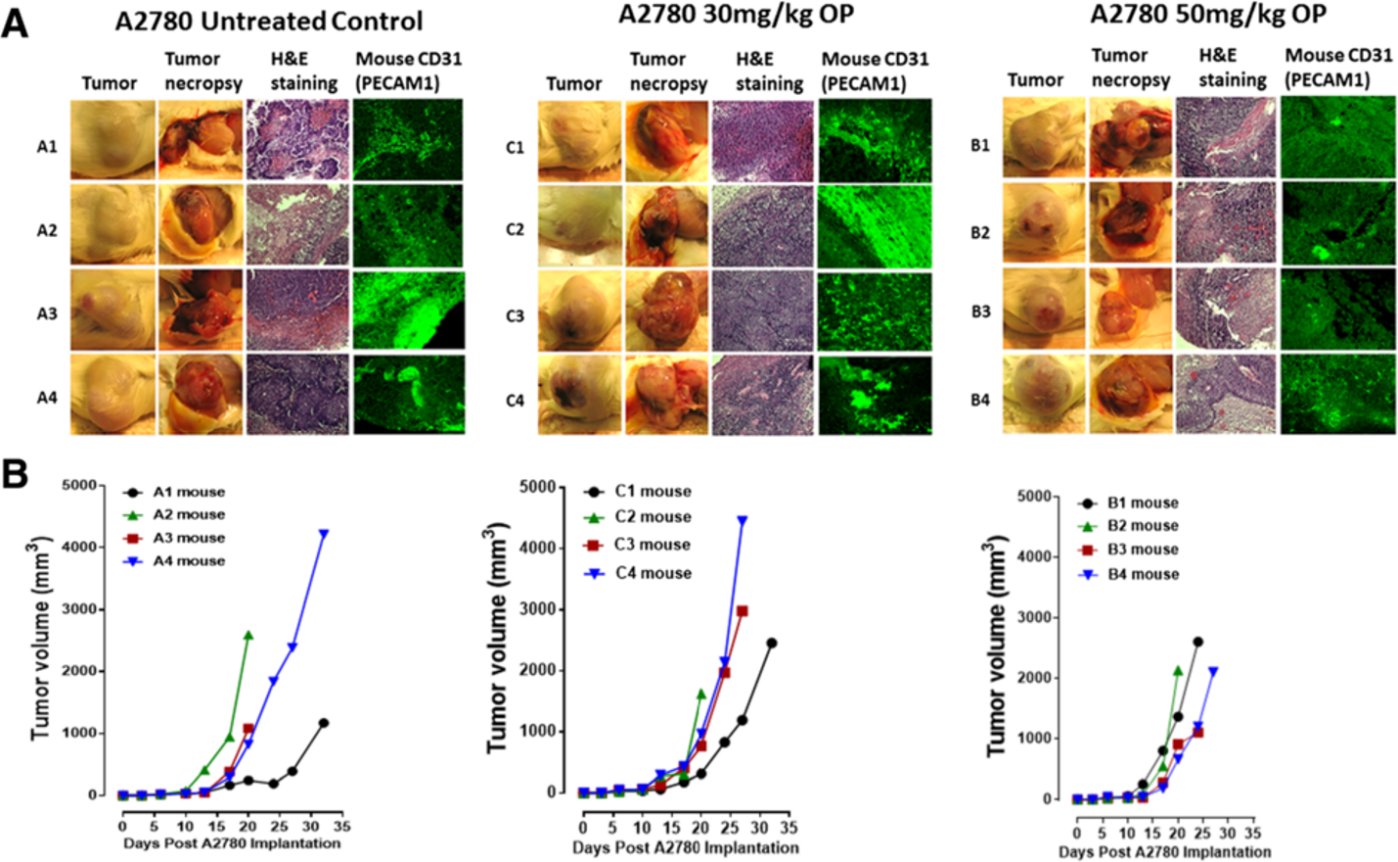
**OP treatment of RAGxCγ double mutant mice bearing heterotopic xenograft of A2780 tumors.** A2780 cells at 0.5 × 10^6^ in 0.2 mL were implanted cutaneously in the right back flank of these mice. Twice a week following implantation of the cancer cells each mouse was monitored for tumor volume ((width square/2) × length) at the site of implantation. Mice were treated with 30 and 50 mg/kg of OP in sterile saline i.p. daily at day 7 post-implantation when the tumor volume reached approximately 50 mm^3^. **(A)** Tumor growth rates for individual mice (mouse label A1-4 for the control cohort; B1-4, 50mg/kg OP cohort and C1-4, 30mg/kg cohort). **(B)** Representative tumors on the right flank of the individual animals at end-point, necropsy of live tumors, H&E staining and immunostaining for mouse CD31+ (PECAM-1) cells of tumor tissues.

The preclinical in vivo anti-tumor activity of OP was investigated in the RAGxCγ double mutant xenograft mouse model of human ovarian A2780 cancer cells. The RAGxCγ double mutant mice lack mature T cells, B cells, and functional NK cells, and are deficient in cytokine signaling, leading to better engraftment of human cells than any other published mouse strain [34]. As previously demonstrated for pancreatic epithelial carcinoma cell line [34], the hypothesis is that OP therapy prevents the in vivo growth and spread of ovarian tumors in the heterotopic xenograft mouse model of human ovarian cancer. A2780 ovarian carcinoma cells at 0.5 × 10^6^ cells in 0.2 mL were implanted cutaneously in the right back flank of these mice. Twice a week, each mouse following implantation of the cancer cells was monitored for tumor volume growth at the site of implantation, body weight, and body condition scoring. All of the experimental mice did not show loss of body weight and body condition scoring was normal until they developed skin lesions. Scoring the body condition of rodents is a non-invasive method for assessing health and establishing endpoints for adults where body weight is not a viable monitoring tool. For heterotopic implantation of A2780 cells, mice developed skin lesions due to the abnormal tumor vascularization, which was the end-point for body condition scoring. The data shown in Figure 4A show (a) live tumors with skin lesions for each mouse (labelled A1-4, B1-4 and C1-4), and (b) necropsy tumors with an unexpected, abnormal robust and bloody tumor vasculature at day 33 or earlier post implantation of A2780 cells. Due to the abnormal tumor vasculature, we also immunostained paraffin-embedded tumors from each mouse for host endothelial cell marker CD31/PECAM-1. The data in Figure 4A show extensive migration of host CD31+ cells in the tumors for the untreated and 30mg/kg OP treated cohorts. For the 50mg/kg OP cohort, there were markedly less host CD31+ cells in the tumor tissues. Treatment with 30 and 50 mg/kg of soluble OP in sterile saline with daily injections intraperitoneally at day 7 post-implantation when the tumor volume reached 50 mm^3^ did not attenuated the aggressive tumor vascularization with skin lesions (Figure 4A), nor the tumor growth (Figure 4B). The treatment regimen with OP was based on previously reported results on pancreatic cancer [34] and unpublished results using breast epithelial triple negative carcinoma. It is proposed that OP treatment regimen for cancers with robust tumor vascularization requires higher doses for attenuating tumor growth as shown by results for pancreatic cancer [34].

**Figure 5:**
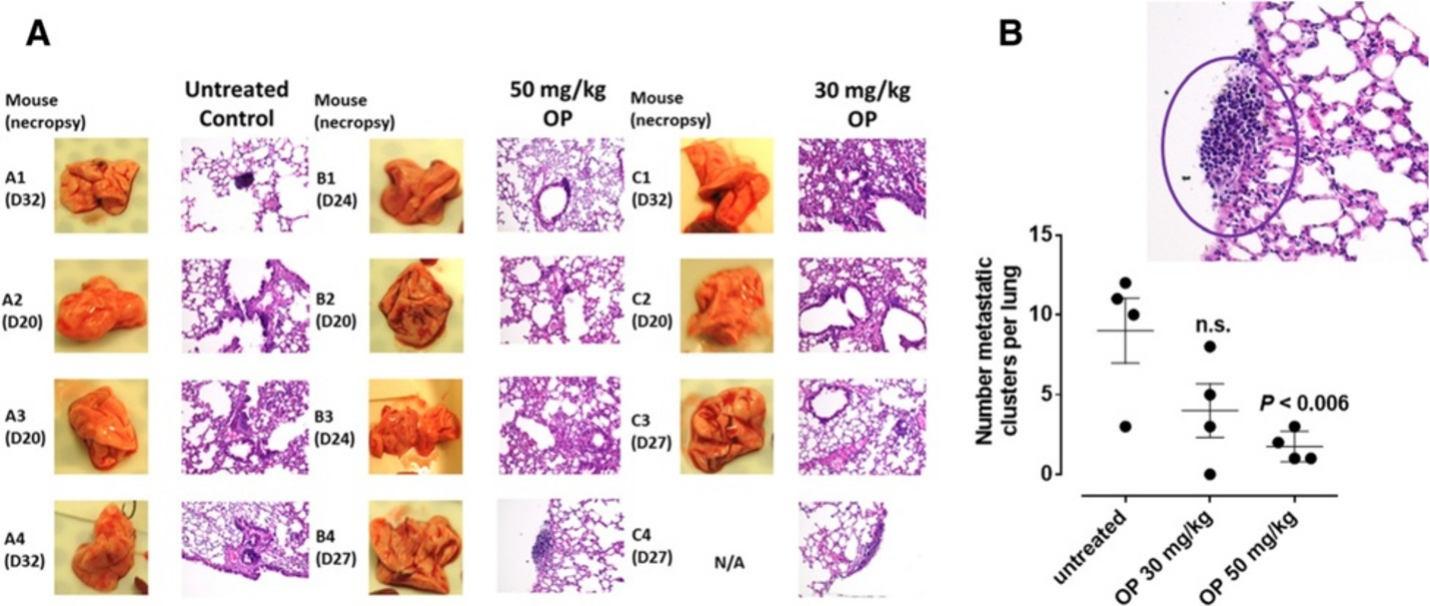
**H&E staining of necropsy lung for number of metastatic clusters per lung in paraffin-embedded tissues taken from xenograft A2780 tumor-bearing RAGxCγ double mutant mice.** **(A)** Mice were implanted with 0.5×106 A2780 cells cutaneously on the rear flank and OP treatment began daily (i.p.) 7 days post implantation when tumors reached 50 mm3. Paraffin-embedded tissue sections (5 μm) on glass slides were processed for H&E staining fir each mouse necropsied at indicated day post-implantation. Stained tissue sections were photographed using AxioCam MRc5 digital color camera attached to a Zeiss Imager M2 fluorescence microscope at 400× magnification. Images are representative of at least five fields of view from two tissue sections. **(B)** Metastatic lung clusters as representative in the insert were microscopically counted per tissues and plotted in the graph. Statistical analysis was carried out using GraphPad Prism and results were compared by unpaired t-test.

Live necropsy lungs (Figure 5A) showed no visible tumor nodules. There were no tumor nodules nor metastatic clusters of cancers cells in the liver for each of the cohorts (data not shown). Surprisingly, the daily dosage of 50 mg/kg OP treatment intraperitoneally significantly attenuated the metastatic spread of ovarian cancer cells to the lung (Figure 5B) compared to the extensive metastatic clusters of cancer cells in the lung for the untreated and the 30mg/kg OP cohorts. This lack of metastatic spread of the ovarian cancer cells to the lung following OP treatment is highly dose dependent on the treatment regime, which may be due to in part of a disruptive tumor vasculature development (reduced host CD31+ endothelial cell migration) or result in a decreased expression of the cell surface mesenchymal marker, N-cadherin, and an increased expression of the cell surface epithelial marker, E-cadherin as previously reported by us for pancreatic cancer [22].

### Silencing transcriptional repressor Snail disables human ovarian carcinoma tumor neovascularization and abolishes heterotopic xenografts of tumor growth and metastasis in RAGxCγ double mutant mice

**Figure 6:**
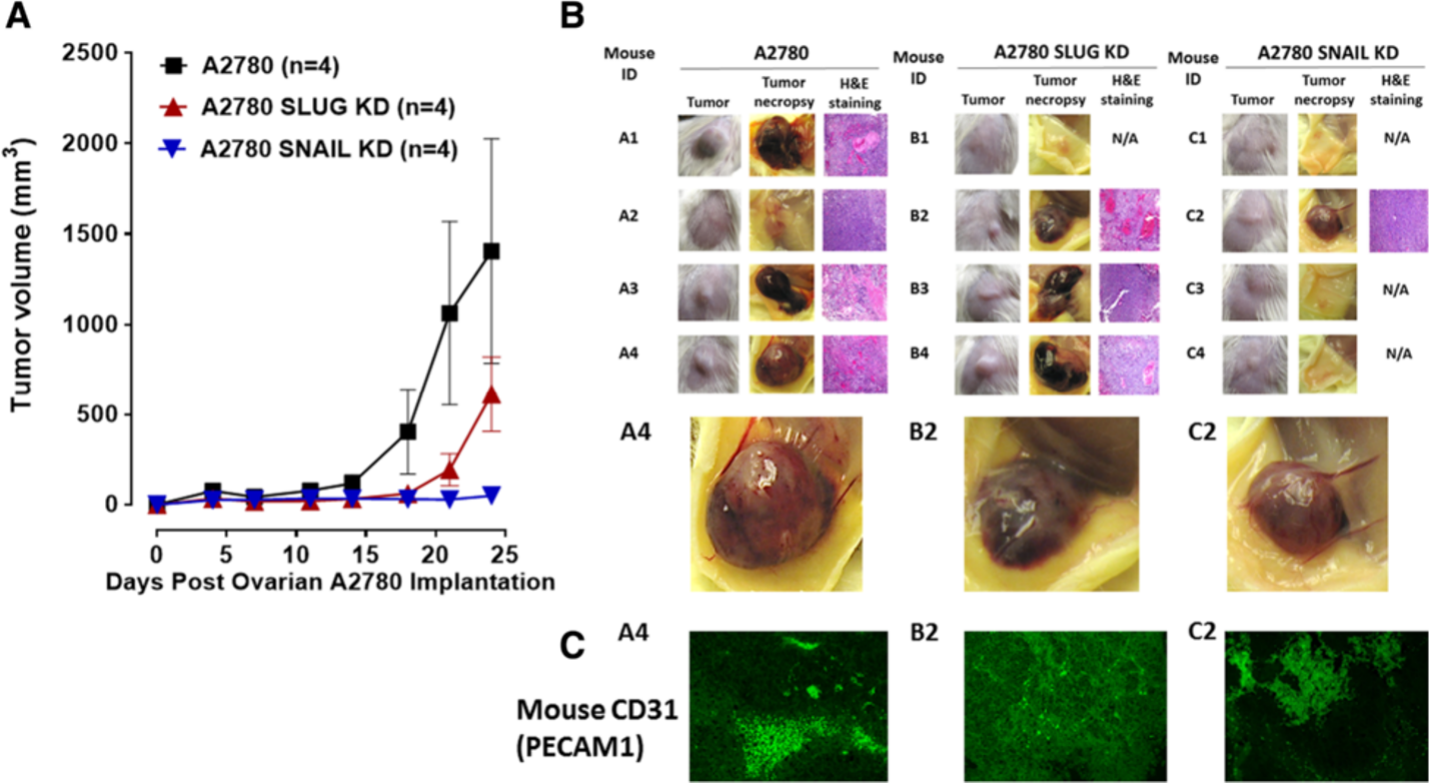
**A2780, A2780 shRNA Snail and A2780 shRNA Slug ovarian cancer cells in heterotopic xenograft of tumors growing in RAGxCγ double mutant mice.** **(A)** Cells at 0.5×106 in 0.2 mL were implanted cutaneously in the right back flank of these mice. Twice a week following implantation of the cancer cells each mouse was monitored for tumor volume ((width square/2) × length) at the site of implantation. Mice were sacrificed at day 24 post-implantation. **(B)** Representative tumors on the right flank of the animal, necropsy tumors and H&E staining of tumors; N/A, not available due to lack of tumor. **(C)** Paraffin-embedded tumor sections (5μm) on glass slides were processed for immunohistochemistry using primary DyLight 488 conjugated rat monoclonal anti-mouse CD31+ (PECAM-1) antibody in 1% BSA in PBS blocking solution and Entellan× rapid mounting media. Stained tissue sections were photographed using an AxioCamMRm3-2 fluorescence camera attached to a Zeiss Imager M2 fluorescence microscope at 400× magnification. Images are representative of at least five fields of view from two tumor sections.

**Figure 7:**
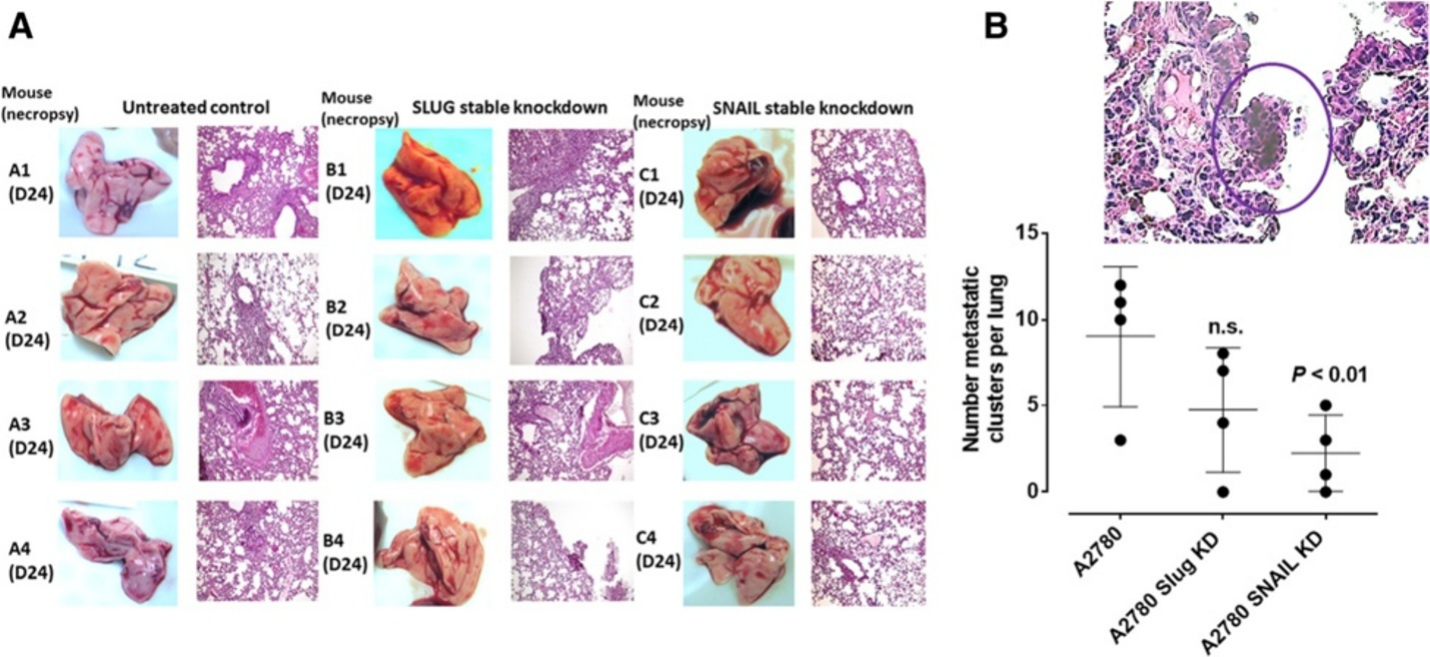
**H&E staining of necropsy lung for number of metastatic clusters per lung in paraffin-embedded tissues taken from xenograft tumor-bearing RAGxCγ double mutant mice.** **(A)** Mice were implanted with 0.5×106 A2780, A2780 shRNA Slug and A2780 shRNA Snail cells cutaneously on the rear flank and OP treatment began daily (i.p.) 7 days post implantation when tumors reached 50 mm3. Paraffin-embedded tissue sections (5μm) on glass slides were processed for H&E staining for each mouse necropsied at indicated day post-implantation. Stained tissue sections were photographed using AxioCam MRc5 digital color camera attached to a Zeiss Imager M2 fluorescence microscope at 400× magnification. Images are representative of at least five fields of view from two tissue sections. **(B)** Metastatic lung clusters as representative in the insert were microscopically counted per tissues and plotted in the graph. Statistical analysis was carried out using GraphPad Prism and results were compared by unpaired t-test.

Although Snail is critical for ovarian cancer growth and metastasis in animal models and human specimens [14],[40], its role in tumor neovascularization remains unknown. Since xenografts of A2780 ovarian tumors produce abnormal robust and bloody tumor vascularization in RAGxCγ double mutant mice (Figure 4B), we investigated whether silencing Snail and its associated member Slug in A2780 cells would attenuate tumor neovascularization in these mice. Using selection media under puromycin, Snail shRNA and Slug shRNA transfected A2780 cell clones revealed a transfection efficiency of 99% stable knockdown as determined using RT-PCR analysis using total RNAs from parental A2780 and Snail and Slug shRNA knockdown cells. The data in Figure 6 clearly demonstrate that silencing Snail but not its Slug associated family member in A2780 cells completely abrogated not only the robust and bloody tumor vascularization (Figure 6B), but also tumor growth (Figure 6A) and metastatic spread to the lung (Figure 7B) in RAGxCγ double mutant mice. There were no metastatic foci in the liver (data not shown).

**Figure 8:**
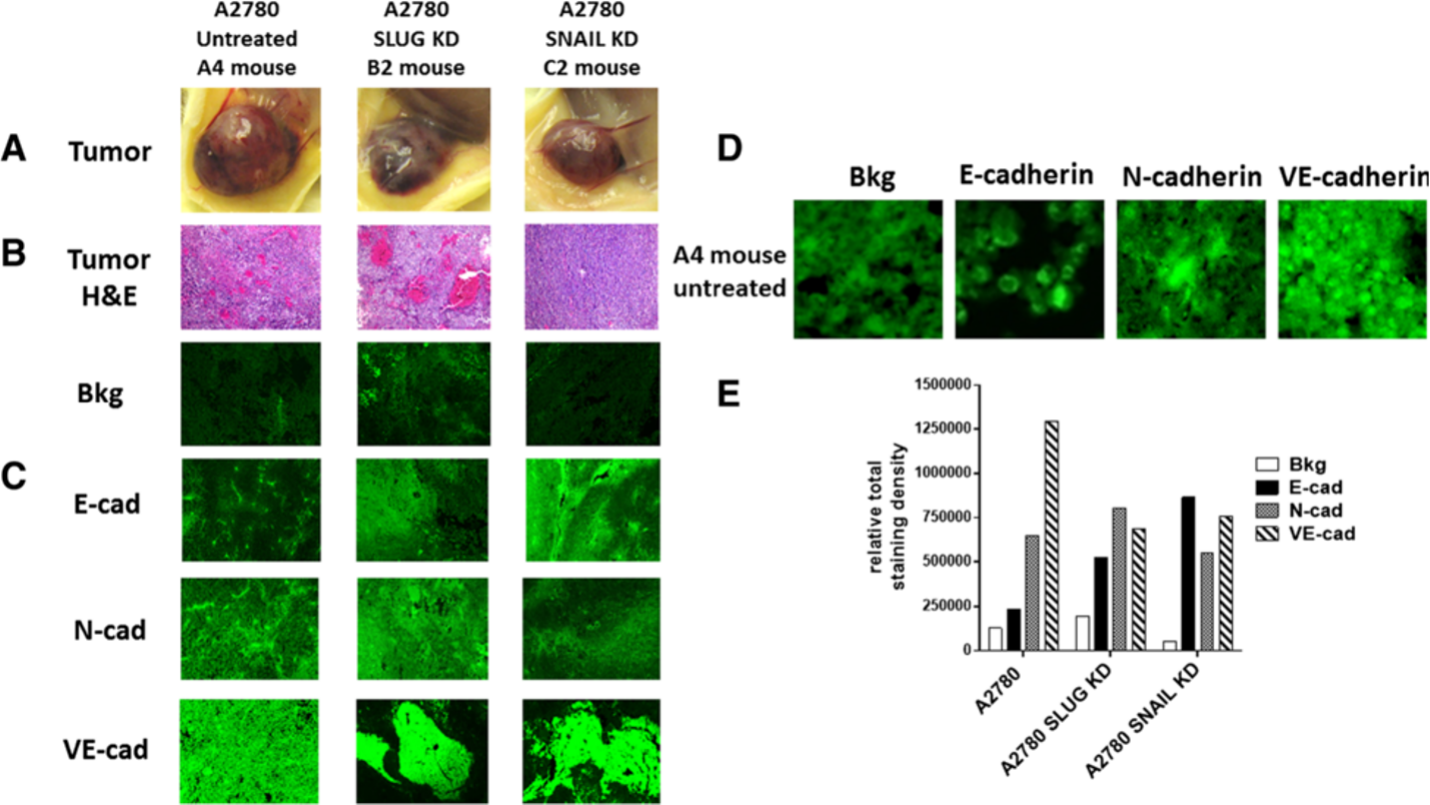
**Fluorescence immunohistochemical detection of E-cadherin, N-cadherin, and VE-cadherin expression in paraffin-embedded tumor tissues archived from xenograft tumors of A2780, A2780 shRNA Snail and A2780 shRNA Slug cells growing in RAGxCγ double mutant mice.** Mice were implanted with 0.5 × 106 cells cutaneously on the rear flank. **(A)** Live necropsy tumors. **(B)** H&E staining of tumor necropsy specimens. **(C)** Paraffin-embedded tumor sections (5 μm) on glass slides were processed for immunohistochemistry using primary anti-E-cadherin, N-cadherin, and VEcadherin antibodies followed with polyclonal goat anti-rabbit Alexa Fluor® 488 secondary antibody and Entellan® rapid mounting media. Background control (Bkg) sections were prepared without the primary antibodies. Stained tissue sections were photographed using an AxioCamMRm3-2 fluorescence camera attached to a Zeiss Imager M2 fluorescence microscope at 200× magnification. Images are representative of at least five fields of view from two tumor sections. **(D)** Tissue sections were visualized and photographed using an AxioCamMRm3-2 fluorescence camera attached to a Zeiss Imager M2 fluorescence microscope at 400× magnification and software enlarged. **(E)** Quantitative analysis was done by assessing the density of tumor staining corrected for background in each panel using Corel Photo Paint 8.0 2 software. Each bar in the figures represents the mean (± standard error of the mean) corrected density of tumor staining within the respective images.

In addition, the A2780 shRNA Snail xenograft tumors (Figure 8A) expressed higher levels of E-cadherin compared to N- and VE-cadherins in contrast to a high expression levels of N- and VE-cadherins in A2780 and A2780 Slug shRNA KD xenograft tumors (Figure 8C,D and E). These findings are consistent with other reports on the role of Snail and E-cadherin in ovarian cancer [40]-[44].

## Discussion

There is substantial evidence to indicate that the zinc-finger transcriptional factors Snail and Slug, the two-handed zinc factors ZEB-1/dEF1 and ZEB-2/SIP1 and the basic helix-loop-helix transcription factors Twist and E12/E47 play major roles in epithelial carcinoma plasticity [2],[15],[41],[42], and in tumor progression and invasiveness [8],[40],[43],[44]. Since Snail is identified as a potent EMT mediator, others have reported that it controls the proteolytic activity of the MMPs that contribute to the phenotypic changes associated with EMT and invasion [40]. Their data indicated that knockdown of Snail expression reduced the mRNA level of MMP-2 and suppressed the gelatinolytic activity of MMP-2 and MMP-9 in vitro, and inhibited the catalytic activity of MMP-2 in vivo. It was proposed that Snail plays an essential role in up-regulating the proteolytic activity of MMPs during invasion and metastasis. Others have provided additional confirmation for Snail in inducing MMP-1, -2, -7 and -14 in liver and squamous cell carcinoma lines [45] as well as MMP-9 in MDCK epithelial cells [14]. However, the molecular mechanism(s) by which the Snail-MMP signaling axis functions in tumor neovascularization remained unknown until now.

Recently, we have reported a novel organizational signaling platform linked to the EGF-induced receptor activation process in live EGFR-expressing cells [34]. This signaling paradigm proposes that EGF binding to its receptor on the cell surface induces a conformational change of the receptor to initiate MMP-9 activation to induce Neu1. Activated Neu1 hydrolyzes α-2,3-sialyl residues linked to β-galactosides, which are distant from the EGF binding sites. These findings predict a prerequisite desialylation process by activated Neu1 enabling the removal of steric hindrance to receptor association which is stabilized by galectin-3. How Neu1 sialidase is rapidly induced by MMP-9 remains unknown. It can be speculated that EGF binding to EGFR on the cell surface initiates a GPCR-signaling via GPCR Gα subunit proteins to activate MMP-9. Our data using co-immunoprecipitation and specific inhibitors of GPCR suggest that the neuromedin B GPCR is associated with EGF-induced sialidase activity in live 3T3-hEGFR cells [34]. Indeed, Moody et al. [46] have also reported that the neuromedin B GPCR regulates EGF receptor transactivation by a mechanism dependent on Src as well as MMP activation. It is well known that agonist-bound GPCRs have been shown to activate numerous MMPs [47], including MMP-3 [48], MMPs 2 and 9 [49],[50], and the members of the ADAM family of metalloproteinases [51],[52]. We have also reported that different GPCR agonists can directly activate Neu1 through the intermediate MMP-9 in order to induce transactivation of TOLL-like receptors and subsequent cellular signaling [28]. It is noteworthy that others have found a dramatic increase in the activity of MMP-9 in gemcitabine-resistant pancreatic cancer cells [53], which fits well within our molecular signaling platform of Neu1-MMP-9 cross-talk in regulating ligand-induced receptor tyrosine kinases.

If MMP-9 is playing a major role in the activation of Neu1 sialidase in complex with EGFR as our data suggest, logistically Snail in inducing MMP-9 in ovarian A2780 cancer cells may be the molecular mechanism(s) by which the Snail-MMP signaling axis functions in tumor neovascularization. Indeed, EGF binding to its receptor on the cell surface of Snail shRNA A2780 cells revealed no Neu1 activity compared to elevated sialidase activity with A2780 and Slug shRNA A2780 cells (Figure 1). OP treatment of A2780, A2780cis, Snail shRNA A2780 and Slug shRNA A2780 cells in culture dose-dependently diminished the cell viability with time. We also observed that OP, anti-Neu1 antibody and specific inhibitor MMP-9i treatment of A2780 ovarian cancer cell line blocked Neu1 activity associated with EGF-stimulation of live A2780 cells. Based on these observations, we propose that Neu1 might be an intermediate candidate connecting the Snail-MMP signaling axis in tumor neovascularization and in promoting the growth and spread of human ovarian cancer.

Clinically, treatment of epithelial ovarian carcinoma is based on the combination of surgery and chemotherapy. Surgical debulking of tumors followed by platinum-based chemotherapy is the standard treatment for advanced ovarian cancer [54]. Unfortunately, the response rates and complete responses in advanced disease are >80% and 40-60%, respectively. After first-line treatment with carboplatin and paclitaxel, most patients eventually relapse with a median progression-free survival of 18 months [55]. Intraperitoneal chemotherapy possibly improves progression-free and overall survivals; however, intraperitoneal chemotherapy has not been universally accepted for the following reasons: toxic effects, intraperitoneal treatment delivery issues and complications [54]. For advanced primary peritoneal carcinoma (PPC) and epithelial ovarian carcinoma, it has been reported that patients receiving primary platinum-taxane chemotherapy do not achieve significant clinical complete responses. PPC patients were found more likely to be platinum resistant at 6 months and had significantly reduced progression-free survival, and overall survival. Indeed, the introduction of novel agents without cross-resistance to platinum or taxanes has been shown to improve the prognosis of platinum-resistant patients [56],[57]. Despite improvements using carboplatin/paclitaxel based chemotherapy, 30% of patients with ovarian cancer fail to respond to primary therapy with 55-75% of responders relapse within 1 or 2 years from the end of primary treatment and die of the disease within 5 years from their initial diagnosis [58]. Alternatively, gemcitabine has been shown to be active as a single agent and in combination with other drugs, including carboplatin and paclitaxel, in the treatment of patients with recurrent ovarian cancer [58]. Unfortunately, other reports have observed different chemotherapeutic complications including hypersensitivity pneumonitis-like patterns in gemcitabine-induced cases [59] and hypersensitivity reactions to oxaliplatin [60]. Based on our present results, it is proposed that ovarian carcinoma treatment regimens could follow a combination therapy of OP at a high dose with low dose of either cisplatin, 5-FU, gemcitabine or paclitaxel. The data in Figure 3 show that for the combination with cisplatin and 5-FU, only OP dosage ≥ 600μg/mL reduced cell viability at 72h compared to monotherapy, while OP did not apparently affect the activity of either gemcitabine or paclitaxel, when compared to the cell viability after single chemo-drug treatment. Based on the fact that hypersensitivity reactions to standard clinical chemotherapeutics are noted complications in cancer patients [59][60], it is proposed from our studies that combination therapy of OP and standard clinical chemotherapeutics may have a beneficial effect. Indeed, we have reported that OP significantly inhibited endotoxin lipopolysaccharide (LPS) induced NFκB activation and the production of nitric oxide and pro-inflammatory IL-6 and TNFα cytokines in primary and macrophage cell lines [31]. It was also shown that primary macrophages obtained from hypomorphic cathepsin A mice with a secondary Neu1 deficiency respond poorly to LPS-induced proinflammatory cytokines [31].

The in vivo anti-tumor activity of OP was also investigated in a RAGxCγ double mutant mouse model of human ovarian cancer. OP therapy at 30 and 50mg/kg daily dosage intraperitoneally did not significantly impeded A2780 tumor growth in a time-to-progression growth rate compared to the untreated cohort. It is noteworthy because of the robust tumor neovascularization that the efficacy of the OP treatment would require a high dose-dependency to ablate the tumor growth. Unexpectedly, the 50 mg/kg OP treatment did cause a significant reduction in the number of metastatic clusters in the lung taken from these tumor-bearing mice (Figure 5B).

Recently, we reported that OP treatment at 100mg/kg of pancreatic tumor-bearing RAGxCγ double mutant mice disrupted the tumor vasculature [34]. Indeed, tumor vasculature requires stringently balanced vascular endothelial growth factor (VEGF) signaling to provide sufficient productive angiogenesis for tumor development. Unexpectedly, the report disclosed an increase in phospho-Smad2-Ser465/467 and phospho-VEGFR2-Tyr1175 in the pancreatic tumor lysates from the OP treated cohort compared to the untreated cohort [34]. Others have found that prolonged VEGF signaling in the absence of endothelial epsins-1 and -2 produces leaky, defective tumor angiogenesis, and thus it was proposed to contribute to the tumor growth retardation [61]. The epsins are a family of ubiquitin-binding endocytic clathrin adaptor proteins that are important in creating membrane curvature. It was shown that epsins-1 and -2 knockout mice exhibited highly disorganized vascular structures with increased vascular permeability in tumors, which was due to an increased VEGFR2 signaling, an increased non-productive tumor angiogenesis and a retarded tumor growth [61]. VEGF binding VEGFR2 promotes epsin binding to ubiquitinated VEGFR2 to facilitate endocytosis and degradation of VEGFR2. It was proposed that epsins-1 and -2 might function as unique attenuators of VEGF signaling [61]. It is possible that OP treatment of ovarian tumor-bearing mice may require stringent OP dose dependency in order to disrupt tumor vasculature, perhaps by blocking epsins as suggested in our previous report [34].

The findings in this report also reveal an unusual robust and bloody tumor vascularization in heterotopic xenografts of A2780 ovarian tumors in RAGxCγ double mutant mice. This immunodeficient mouse model has a double mutation in the combining recombinase activating gene-1 (RAG1) and common cytokine receptor γ chain (Cγ) [35]. The RAGxCγ double mutant mice on a Nod genetic background are completely alymphoid (T- and B-lymphocyte, and NK-cell deficient), show no spontaneous tumor formation, and exhibit normal hematopoietic parameters. When Snail shRNA A2780 cells were injected cutaneously, the xenografts of tumors showed no robust bloody tumor vascularization compared with A2780 and A2780 shRNA Slug tumors, and the mice were completely devoid of tumor growth and metastatic spread to the lungs. Our observations and other reports have shown that the knockdown of Snail expression not only suppressed ovarian cancer metastasis but also inhibited primary tumor growth [40]. Our data also indicate that Snail not only plays a dual role in controlling the growth and metastasis of ovarian cancer, but also functions as a mediator of tumor neovascularization.

Surprisingly, we found that xenografts of Slug shRNA A2780 tumors also revealed robust bloody tumor vasculature similar to the A2780 tumors with no significant inhibition of tumor growth and metastasis to the lungs. To explain these findings, other reports have shown that the transcriptional repressor Slug is a highly unstable protein [44],[62], whereas the expression of Snail plays a more dominant role. It has been reported that the partner of paired (Ppa) protein, an F-box-containing a modular component of an E3 ubiquitin ligase, binds to the Slug protein and promotes its degradation [62]. In other reports, Slug by itself is incapable of triggering EMT as a stable, long-term process, but requires the expression of Snail for a stable down-regulation of E-cadherin [44]. In contrast, another report suggested that Slug triggers EMT independent of Snail expression [42]. To explain the dichotomy role of Slug in mediating EMT, it was proposed that Slug has a surveillance role in rapidly signaling the onset of adverse conditions which may be evaded by rapid migration of cells to a niche more favorable for growth through Snail mediated EMT [44]. Alternatively, the high endogenous levels of Slug expression may promote tumorigenesis through the acquisition of resistance to apoptosis [63]. For tumor neovascularization, silencing Slug in ovarian A2780 cells did not prevent the robust vascularization in RAGxCγ double mutant mice, whereas silencing Snail in A2780 cohort had a profound complete inhibitory effect. Collectively, these findings with other reports suggest that the signaling factors in the pathways that regulate the expression of the Snail and Slug proteins during different developmental processes are tissue- and organism-dependent [64]. For an example, Snail has been shown to be expressed in renal cell carcinomas (RCCs) with positive associations with primary tumor stage and nuclear grade [43]. The report found that Slug expression is negatively associated with primary tumor stage, suggesting the down-regulation of Slug expression during malignant progression of RCCs. Silencing Snail with small interfering RNA (siRNA) led to the down-regulation of MMP-2 and MMP-9, and up-regulation of E-cadherin together with inhibition of the cell invasion through Matrigel in vitro, whereas siRNA for Slug showed no such effects. Jin et al. have also demonstrated that Snail-specific antisense constructs blocked migration and invasion of ovarian cancer cells by restoring E-cadherin expression [40]. Other reports have shown that (a) the E-cadherin repressor Snail is associated with cancer invasion and prognosis [43] and (b) overexpression of Snail in epithelial MDCK cells promotes the epithelial to mesenchymal transition (EMT) towards the invasive phenotype [14]. The expression of Snail in epithelial MDCK cells induced an increase in the promoter activity and expression of MMP-9 through the PI3K/MAPK signaling pathway in these cells. For A2780 ovarian cancer cells, our data provide additional confirmation for Snail's role in tumor neovascularization, the mechanism(s) of which may involve the Snail-MMP-9 signaling axis in facilitating the signaling paradigm for the activation of growth factor tyrosine kinase receptors through a GPCR-signaling process and MMP-9 activation to induce Neu1 as previously reported by us [33].

## Conclusion

**Figure 9:**
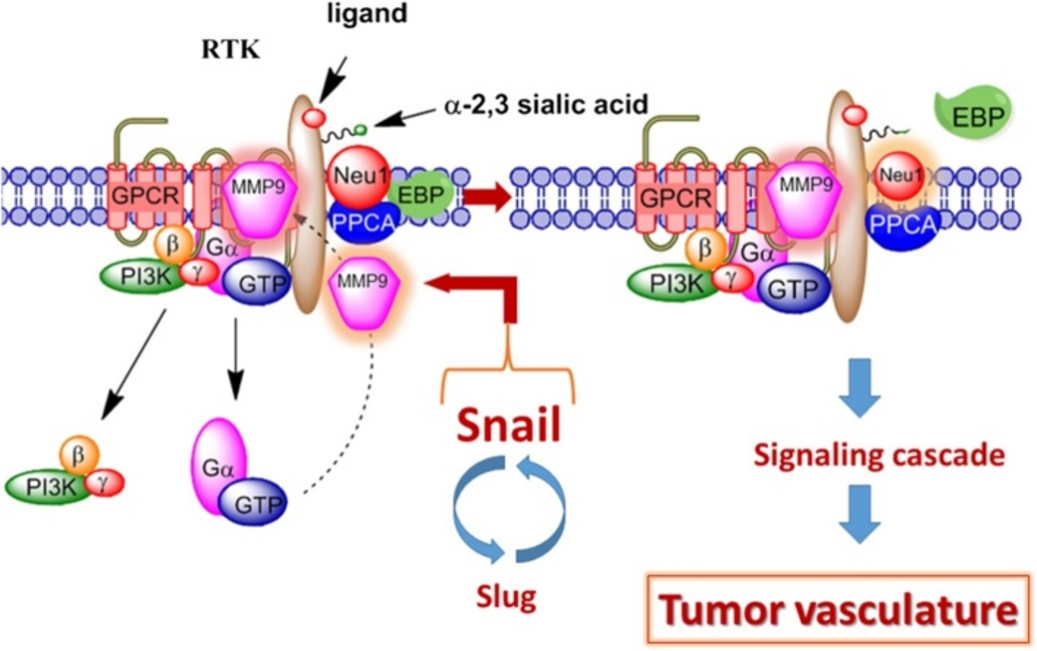
**Graphical abstract of the Snail and MMP-9 signaling axis in facilitating a neuraminidase-1 (Neu1) and matrix metalloproteinase-9 (MMP-9) cross-talk in regulating receptor tyrosine kinases (RTKs) in ovarian cancer cells to promote tumor neovascularization.** Notes: For ovarian cancers, Snail and MMP-9 expressions are closely connected in similar invasive tumor processes. Snail induces MMP-9 secretion via multiple signaling pathways, but particularly in cooperation with oncogenic H-Ras (RasV12), Snail leads to the transcriptional up-regulation of MMP-9. This Snail-MMP-9 signaling axis is the connecting link in promoting growth factor receptor glycosylation modification involving the subsequent receptor-signaling platform of a Neu1-MMP-9 crosstalk tethered at the ectodomain of RTKs. Activated MMP-9 is proposed to remove the elastin-binding protein (EBP) as part of the molecular multi-enzymatic complex that contains β-galactosidase/Neu1 and protective protein cathepsin A (PPCA). Activated Neu1 hydrolyzes α-2,3-sialic acid residues of RTKs at the ectodomain to remove steric hindrance to receptor association and activation. This process sets the stage for Snail's role in tumor neovascularization. Abbreviations: GPCR, G-protein coupled receptor; Pi3K, phosphatidylinositol 3-kinase; GTP, guanine triphosphate; EBP, elastin binding protein; PPCA, protective protein cathepsin A. Citation: Taken in part from Research and Reports in Biochemistry 2013:3 17-30. © 2013 Abdulkhalek et al, publisher and licensee Dove Medical Press Ltd. This is an Open Access article which permits unrestricted non-commercial use, provided the original work is properly cited.

The results of this study are the first to show the ability of transcriptional factor Snail in mediating ovarian tumor neovascularization. Silencing transcriptional factor Snail in A2780 ovarian carcinoma cells ablated the abnormal robust and bloody tumor vascularization in RAGxCγ double mutant mice with a concomitant abolishment of tumor growth and metastatic spread to the lungs. For invasive tumors like ovarian cancers, Snail and MMP-9 expressions are closely connected since they have both been implicated in similar invasive processes [65]. Moreover, it has been shown that Snail induces MMP-9 secretion via multiple signaling pathways, but particularly in cooperation with oncogenic H-Ras (RasV12), Snail leads to the transcriptional up-regulation of MMP-9 [14]. Collectively, these different signaling paradigms involved with EMT in ovarian cancer suggest that growth factor receptor glycosylation modification involving the receptor-signaling platform of a Neu1-MMP-9 crosstalk may in fact be the invisible link connecting the Snail-MMP-9 signaling axis. It follows that the therapeutic efficacy of oseltamivir phosphate targeting Neu1 tethered to these receptors would be critically dose dependent. Given the ability of OP to increase E-cadherin expression and decrease N-cadherin and VE-cadherin expression as previously reported by us [22], ovarian cancer treated with this drug may become more adherent to the surrounding tissue and not metastasize as our data indicate. We propose here a graphical abstract illustrating that the Snail-MMP-9 signaling axis maintains several important cancer growth factor receptor signaling platforms in promoting Neu1-MMP-9 crosstalk in complex with these receptors (Figure 9). Activated Neu1 hydrolytically cleaves α-2,3-linked sialic acid(s) from the receptor's ectodomain, the desialylation process allowing for the removal of steric hindrance and thus for proper receptor dimerization and activation of its signaling cascade networks within the cancer cell. OP treatment strategies under dose dependence is proposed to take the form of a horizontal approach, of which, different oncogenic signaling pathways and macrophage-mediated tumorigenesis are targeted with promising therapeutic intent.

## Authors' contributions

SA and MR wrote the paper, designed and performed experiments; SA did the A2780 shRNA Snail and Slug pGIZ lentiviral transfections; FA and SA performed the sialidase assay; LB and FH performed the WST assay; OG and DS performed the liver and lung metastatic tumor analyses; OG performed the immunohistochemistry; FH and LO did the immunostaining for host CD31+ cells in tumor tissues; MRS supervised the research design and the writing of the paper. All authors read and commented on the manuscript.

## Authors' information

SA is a recipient of the RSM scholarship, the Ontario Graduate scholarship, and the Canadian Institutes of Health Research Doctoral award (Frederick Banting and Charles Best Canada Graduate scholarship). OG was the recipient of the 2013 NSERC USRA. FA was the recipient of the King Abdullah Scholarship from the Ministry of Higher Education, Saudi Arabia.

## Authors’ original submitted files for images

Below are the links to the authors’ original submitted files for images. Authors’ original file for figure 1 Authors’ original file for figure 2 Authors’ original file for figure 3 Authors’ original file for figure 4 Authors’ original file for figure 5 Authors’ original file for figure 6 Authors’ original file for figure 7 Authors’ original file for figure 8 Authors’ original file for figure 9

## Abbreviations

OP: Oseltamivir phosphate
EMT: Epithelial to mesenchymal transition
TGF-β: Transforming growth factor-β
TLR: TOLL-like receptor
MMP: Matrix metalloproteinase
H&E: Hematoxylin and eosin
i.p.: Intraperitoneally
EGF: Epidermal growth factor
MMP-9i: Specific inhibitor of MMP-9
LD_50_: Individual dose required to kill 50 percent of viable cells
5-FU: 5-Fluorouracil
Bkg: Background
cad: Cadherin
