# Supplementary figures and images for: Transcriptional factor snail controls tumor neovascularization, growth and metastasis in mouse model of human ovarian carcinoma
Source: Clin Transl Med. 2014 Sep 23;3:28. doi: 10.1186/s40169-014-0028-z (PMC4884043; doi:10.1186/s40169-014-0028-z)

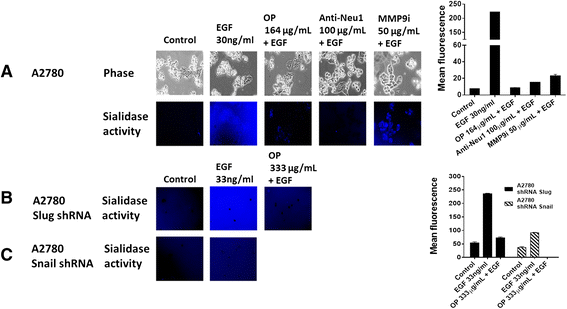

Supplement: Supplementary file 1 — Authors’ original file for figure 1 [file 40169_2014_28_MOESM1_ESM.gif]

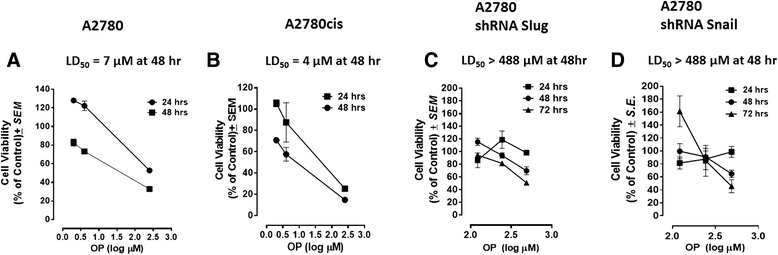

Supplement: Supplementary file 2 — Authors’ original file for figure 2 [file 40169_2014_28_MOESM2_ESM.gif]

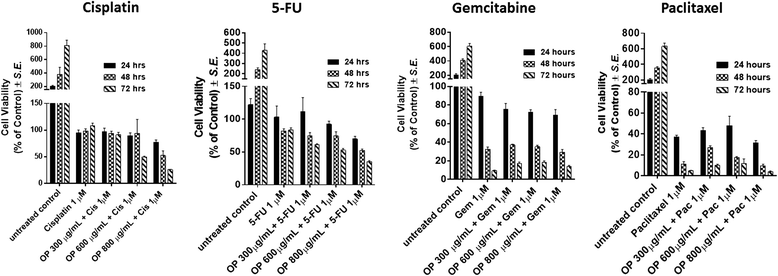

Supplement: Supplementary file 3 — Authors’ original file for figure 3 [file 40169_2014_28_MOESM3_ESM.gif]

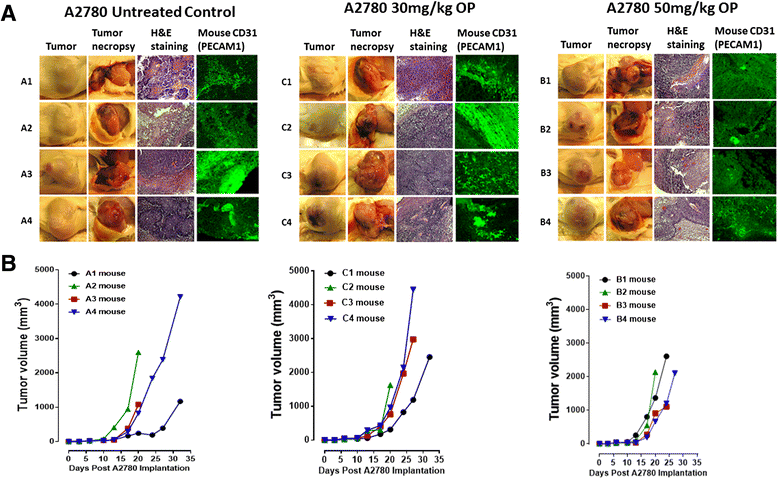

Supplement: Supplementary file 4 — Authors’ original file for figure 4 [file 40169_2014_28_MOESM4_ESM.gif]

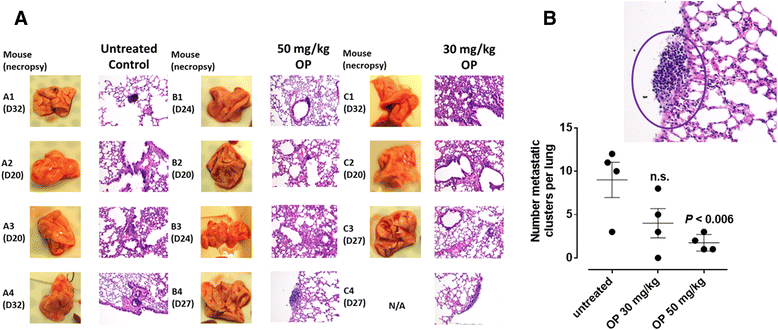

Supplement: Supplementary file 5 — Authors’ original file for figure 5 [file 40169_2014_28_MOESM5_ESM.gif]

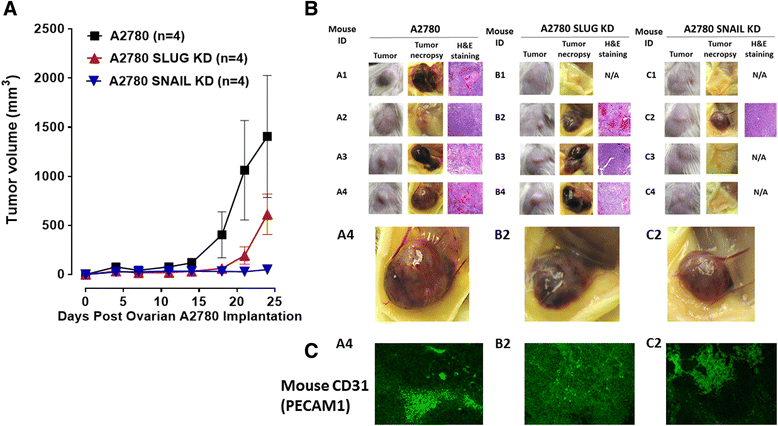

Supplement: Supplementary file 6 — Authors’ original file for figure 6 [file 40169_2014_28_MOESM6_ESM.gif]

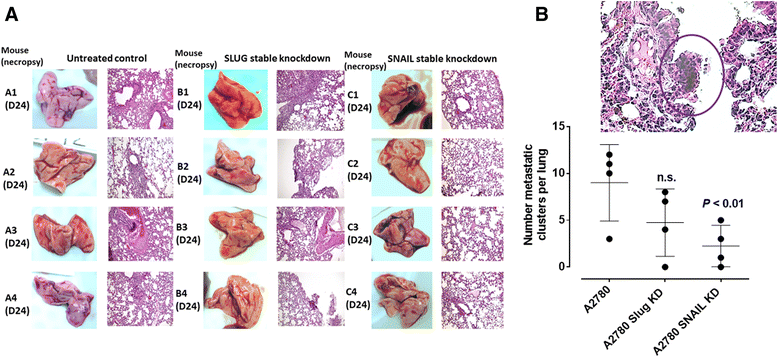

Supplement: Supplementary file 7 — Authors’ original file for figure 7 [file 40169_2014_28_MOESM7_ESM.gif]

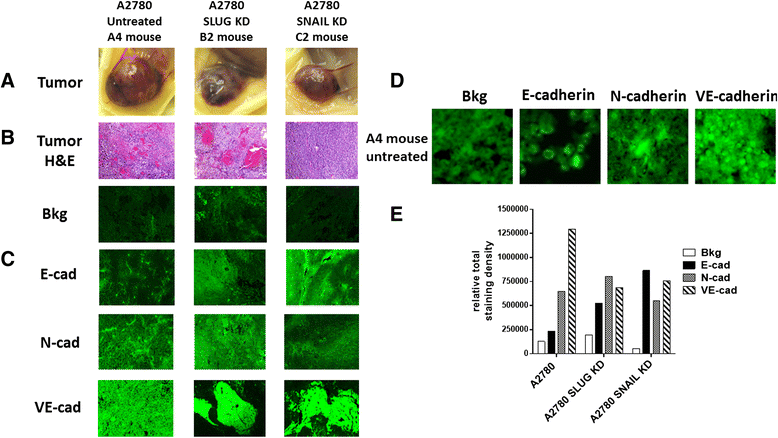

Supplement: Supplementary file 8 — Authors’ original file for figure 8 [file 40169_2014_28_MOESM8_ESM.gif]

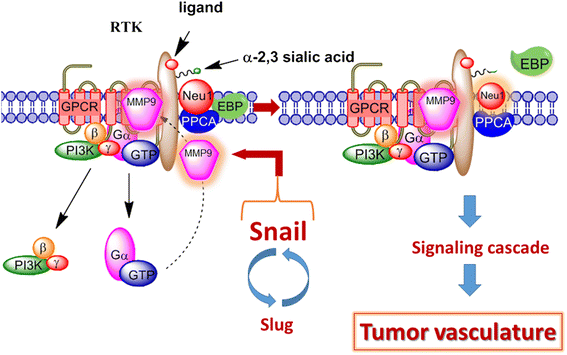

Supplement: Supplementary file 9 — Authors’ original file for figure 9 [file 40169_2014_28_MOESM9_ESM.gif]
